# Lights and Shadows on the Sourcing of Silver Radioisotopes for Targeted Imaging and Therapy of Cancer: Production Routes and Separation Methods

**DOI:** 10.3390/ph16070929

**Published:** 2023-06-26

**Authors:** Marianna Tosato, Mattia Asti

**Affiliations:** Radiopharmaceutical Chemistry Section, Nuclear Medicine Unit, AUSL-IRCCS di Reggio Emilia, 42122 Reggio Emilia, Italy

**Keywords:** radioisotopes, theranostic, silver, cancer

## Abstract

The interest in silver radioisotopes of medical appeal (silver-103, silver-104m,g and silver-111) has been recently awakened by the versatile nature of their nuclear decays, which combine emissions potentially suitable for non-invasive imaging with emissions suited for cancer treatment. However, to trigger their in vivo application, the production of silver radioisotopes in adequate amounts, and with high radionuclidic purity and molar activity, is a key prerequisite. This review examines the different production routes of silver-111, silver-103 and silver-104m,g providing a comprehensive critical overview of the separation and purification strategies developed so far. Aspects of quality (radiochemical, chemical and radionuclidic purity) are also emphasized and compared with the aim of pushing towards the future implementation of this theranostic triplet in preclinical and clinical contexts.

## 1. Introduction

When properly harnessed, the radiation emitted during the decay of unstable nuclei can be exploited for the diagnosis and treatment of cancer. In recent years, cutting-edge studies in this field delved into the exploration of new metallic radionuclides able to provide a broad plethora of diagnostic and therapeutic emissions, but also the new challenges in their production and purification.

Among the metals of interest, silver exhibits a vast series of radioisotopes whose half-lives range from minutes to a few days, covering almost all the decay modes except for α-emission. Within the silver radioisotopes of potential medical relevance, silver-111 (^111^Ag) is a medium energy *β*^−^ emitter (*E_β_*^−^_, max_ = 1.04 MeV), with a 7.47 d half-life (*t*_1/2_), that could be harnessed for the treatment of medium to large tumors due to its particulate emission ranging from one to ten millimeters [1,2]. The direct decay of ^111^Ag to cadmium-111 (^111^Cd) ground state, is also accompanied by the emission of several γ-rays. One of them, with 342.1 keV energy and 6.7% abundance, can potentially enable the visualization of the radiometal biodistribution via single-photon emission computed tomography (SPECT) during the therapy follow-up [1]. It is interesting to underline the notable similarity of ^111^Ag properties (i.e., emissions and half-life) with the lutetium-177 ones (^177^Lu, *t*_1/2_ = 6.67 days, *E_β_*^−^_,max_ = 0.498 MeV, *E*_γ_ = 208 keV, 11% abundance), ^177^Lu currently being the most prominent therapeutic isotope. This equivalence makes ^111^Ag a potential ^177^Lu surrogate and could pave the way for the subsequent development of ^111^Ag-labelled radiopharmaceuticals.

Other medically appealing silver radioisotopes include the positron (*β*^+^) emitters silver-103 (^103^Ag, *t*_1/2_ = 65.7 min) and silver-104m,g (^104m^Ag, *t*_1/2_ = 33.5 min and ^104g^Ag, *t*_1/2_ = 69.2 min, respectively) that could be paired with ^111^Ag to perform positron emission tomography (PET) diagnosis and pre-therapy selection, thus enabling steps towards personalized medicine [3,4]. The main decay properties of ^103^Ag, ^104m,g^Ag and ^111^Ag are summarized in Table 1.

Despite the theoretical suitability of silver radioisotopes for cancer imaging and therapy, their application for nuclear medicine purposes has been mostly ignored so far, mainly because of the difficulties related to their production and the challenges embedded in their separation from target materials. Moreover, the complexation chemistry of silver is an almost uncharted field, apart from a few groundbreaking investigations [5,6,7]. As a result, there are no reports on preclinical nor clinical studies based on silver radioisotopes to date, except those related to the use of ^111^Ag-labelled nanoparticles as antimicrobial agents or ^111^Ag-labelled hydroxyapatite for radiosynovectomy, respectively [8,9,10,11].

The present review summarizes the state-of-the-art in the production routes applied or postulated to supply these three medically interesting radioisotopes in a sufficiently high molar activity and radionuclidic purity, as well as it revises the purification strategies developed up to now in order to achieve a proper separation of silver radioisotopes from the target matrix and the co-produced elements. The aim of this overview is to foster the future implementation of the silver theranostic triplet in preclinical and clinical contexts.

**Table 1:** Decay properties of silver radioisotopes for diagnostic and therapeutic applications from IAEA database [12] *.

Radioisotope	Use	*t*_1/2_	Decay Mode	*E_β_*^−^ [keV]	*I_β_*^−^ [%]	*E_β_*^+^ [keV]	*I_β_*^+^ [%]	*E*_γ_ [keV]	*I*_γ_ [%]
^103^Ag	PET	65.7 min	*ε* + *β*^+^ (100%)	*-*	*-*	2421	14.8	118.7	31.2
						2688	8	148.2	28.3
						2444	2.1	266.9	13.3
						2156	1.6	1273.8	9.4
^104g^Ag	PET	69.2 min	*ε* + *β*^+^ (100%)	*-*	*-*	2014	5.1	555.8	92.6
						2197	4.1	767.6	65.7
						2097	2.9	941.6	25.0
						2955	2.0	925.9	12.5
^104m^Ag	PET	33.5 min	*ε* + *β*^+^ + IT (100%)			3730	58.0	555.8	91
				*-*	*-*	2492	1.8	1341.7	1.6
						2944	1.1	767.6	0.9
^111^Ag	Therapy (+ SPECT)	7.4 d	*β*^−^ (100%)	1035	92.0	*-*	-	342.1	6.7
				694.7	7.1			245.4	1.2
				791.4	1.0				

* Only decays with the highest abundance are reported.

## 2. Production of Silver-111

Several nuclear reactions that produce ^111^Ag have been reported to date, ranging from thermal neutron irradiation to photonuclear-based production. These production routes are summarized and critically analyzed in the following paragraphs.

### 2.1. Reactor-Based Production

^111^Ag can be obtained by thermal neutron irradiation of a palladium target via the ^110^Pd(n,γ)^111^Pd nuclear reaction and by the subsequent decay of the intermediate nuclide palladium-111 (^111^Pd, *t*_1/2_ = 23.4 min) to ^111^Ag [13,14]. Natural palladium might be used for this approach but, in this case, several additional radionuclides are concurrently produced along with ^111^Ag due to the parallel parasitic reactions occurring on the other isotopes composing the natural element. Consequently, ^111^Ag produced from the neutron irradiation of natural palladium targets is always a marginal product of the reaction and it is contaminated with several stable and radioactive nuclides, including silver isotopes themselves. For the sake of clarity, palladium isotopes’ natural abundances and (radio)nuclides derived from neutron irradiation, along with the corresponding cross-section (σ) values, are summarized in Table 2. The presence of additional radioactive species, such as palladium-103 (^103^Pd) and palladium-109 (^109^Pd), largely decreases the radionuclidic purity of ^111^Ag and hence its applicability as a medical radioisotope [13]. For example, Alberto et al. reported the production of 100 MBq of ^111^Ag and 1630 MBq of ^109^Pd when irradiating 100 mg of natural palladium for 26 h (followed by 72 h of cooling to reduce ^109^Pd activity) at a neutron flux of 3 × 10^13^ n/cm^2^/s [13]. On the other hand, the co-production of stable Ag isotopes which cannot be chemically separated from ^111^Ag—i.e., silver-107 (^107^Ag) and silver-109 (^109^Ag)—dwindles the molar activity of the desired radionuclide, hampering the radiolabeling of tumor-targeting vectors. Indeed, the stable Ag impurities compete in the binding with the same affinity of ^111^Ag and this feature could result in an ineffective diagnostic and/or therapeutic outcome since the excess of unlabeled bioconjugate, needed for providing quantitative labeling, may saturate the target receptors on cancer cells.

The use of ^110^Pd-enriched targets is thus recommended to improve ^111^Ag yield and circumvent the parasitic reactions summarized in Table 2. For instance, Morselli et al. recently reported that the neutron irradiation of 100 mg of enriched palladium for 3 d (6 h/d) at a neutron flux of 1.7 × 10^13^ n/cm^2^/s, followed by 18 h of cooling, yields 102 MBq of ^111^Ag and only 30 MBq of ^109^Pd (due to the low abundance impurity of palladium-108 in the target) [15]. Unfortunately, even if the presence of silver stable isotopes is largely avoided, the use of an enriched target does not overcome the need for chemical purification after the irradiation to remove the metal impurities generated in the process and recover the target material. The high cost of such material requires, indeed, the establishment of extremely efficient recycling strategies to recover the ^110^Pd for further irradiations.

### 2.2. Accelerator-Based Production

#### 2.2.1. Charged-Particle-Based Production

**Figure 1:**
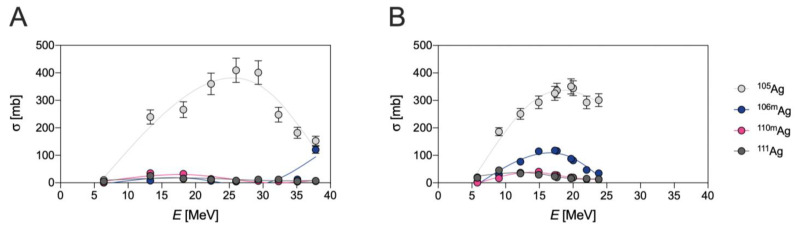
Experimental cross-sections of deuteron-induced ^nat^Pd(d,*x*)^105^Ag, ^106m^Ag, ^110m^Ag and ^111^Ag reactions, as derived from (**A**) Ditroi et al. and (**B**) from Ukon et al. [4,17].

The production of ^111^Ag can also be achieved by deuteron-induced reactions via the ^110^Pd(d,n)^111^Ag direct pathway [3,14,16] or via the ^110^Pd(d,p)^111^Pd → ^111^Ag indirect route [14,17]. In addition to ^111^Ag, the irradiation of natural palladium produces several silver isotopes such as ^103^Ag, ^104^Ag, silver-105m,g (^105g^Ag, *t*_1/2_ = 41.3 d; ^105m^Ag, *t*_1/2_ = 7.2 min), silver-106m (^106m^Ag, *t*_1/2_ = 8.3 d) and silver-110m (^110m^Ag, *t*_1/2_ = 249.8 d) in the direct route [17] and stable ^109^Ag in the indirect route, respectively [14,17]. In fact, ^109^Ag is derived from the decay of ^109^Pd that is, in turn, generated by the ^108^Pd(d,p)^109^Pd and ^110^Pd(d,p2n)^109^Pd reactions. A summary of the cross-sections to produce all the silver isotopes herein reported, via deuteron-based reactions on the natural palladium targets, is reported in Figure 1.

As can be observed, the production of ^111^Ag in these conditions is a mere side-nuclear reaction, occurring with a very low cross-section.

**Figure 2:**
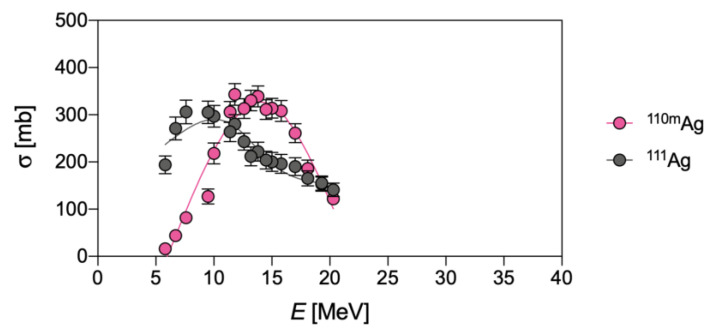
Experimental cross-sections of deuteron-induced ^110^Pd(d,n)^111^Ag and ^110^Pd(d,2n)^110m^Ag reactions, as derived from Hermanne et al. [3].

Due to their short half-life, ^103^Ag and ^104^Ag would not pose an unsurpassed issue for the radionuclidic purity of ^111^Ag, since ^103^Pd (*t*_1/2_ = 17.0 d, decay mode: electron capture—EC and *β*^+^ emission to stable rhodium-103—^103^Rh) and stable palladium-104 (^104^Pd)—generated by EC and *β*^+^ emission from ^103^Ag and ^104^Ag, respectively—can be removed during the subsequent chemical processing. Contrarily, ^105^Ag (decay mode: EC and *β*^+^ emission to palladium-105—^105^Pd), ^106m^Ag (decay mode: EC and *β*^+^ emission to palladium-106—^106^Pd) and ^110m^Ag (decay mode: internal transition (IT) to ^110^Ag and *β*^−^ emission to stable cadmium-110—^110^Cd) and stable ^109^Ag possess longer half-lives than ^111^Ag, and cannot be removed by chemical methods. As a result, the irradiation of a ^110^Pd-enriched target is necessary but, unfortunately, not sufficient to obtain carrier-free ^111^Ag, since significant amounts of ^110m^Ag are also co-produced through the ^110^Pd(d,2n)^110m^Ag reaction. Cross-sections for ^110m^Ag and ^111^Ag production on a ^110^Pd-enriched target are compared in Figure 2.

Finally, even if the yield of the direct pathway on a ^110^Pd-enriched target is rather high for a charged particle reaction (i.e., 4 GBq/C at 20 MeV, target thickness 500 mg/cm^2^, 200 μA/1.3∙10^15^ particles/s), this route is rather unfavorable when compared to the neutron-based production (vide supra) [3]. In fact, although the cross-sections of the two pathways are roughly comparable (compare Table 2 with Figure 2), the neutron flux can be much higher than the impacting deuterons, leading, consequently, to a corresponding higher activity per unit amount of target material.

**Figure 3:**
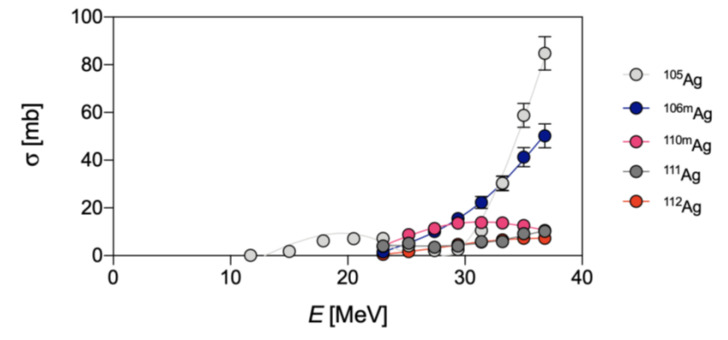
Experimental cross-sections of the α-induced reactions ^nat^Pd(α,p*x*n)^105^Ag, ^106m^Ag, ^110m^Ag, ^111^Ag and ^112^Ag, as derived from Hermanne et al. [18].

The α-induced reactions, ^108^Pd(α,p)^111^Ag and ^110^Pd(α,p2n)^111^Ag, could also be harnessed to produce ^111^Ag using palladium targets. However, the low cross-sections, summarized in Figure 3, render these production routes unworthy alternatives compared with the previously described ones, even when highly enriched target materials are used [18].

**Figure 4:**
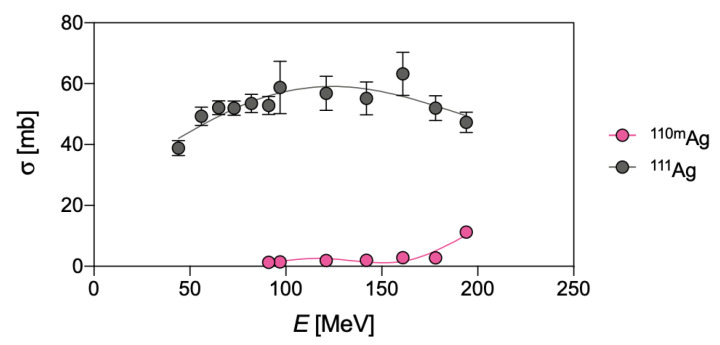
Experimental cross-sections of the proton-induced reactions, ^232^Th(p,f)^111^Ag and ^232^Th(p,f)^110m^Ag, as derived from Mastren et al. [1].

^111^Ag can also be obtained as a secondary product in the process originally devoted to the production of the medically interesting α-emitter actinium-225 (^225^Ac, *t*_1/2_ = 9.9 d), i.e., through the proton irradiation of a thorium matrix [1]. According to the cross-section data reported in Figure 4, a production of around 518 GBq of ^111^Ag is predictable by the ^232^Th(p,f)^111^Ag reaction, when massive thorium targets (100 g) are bombarded with 90 MeV incident proton at 200 μA intensity during a full-scale production of ^225^Ac at Los Alamos National Laboratories [1]. This dual-production method has the potentially huge benefit of concomitantly providing ^111^Ag along with ^225^Ac in a single run, thus reducing the costly time of irradiation in comparison with two independent productions of the single radionuclides. However, it exhibits an unsubtle drawback in the time-consuming separation of ^111^Ag from the target matrix and from the several radionuclides produced during the fission reactions (vide infra). Moreover, the presence of long-lived ^110m^Ag (*t*_1/2_ = 249.8 d), as a by-product of the ^232^Th irradiation (Figure 4), is an additional concern and preclinical studies are warranted to examine and clarify the dosimetry derived from the injection of subtle amounts of this impurity [1].

#### 2.2.2. Photonuclear-Based Production

The possibility to obtain ^111^Ag on natural cadmium targets via the ^112^Cd(γ,p)^111^Ag pathway has also been recently reported [14]. However, simultaneous (γ,p)-reactions with comparable energy thresholds, induced on the isotopes composing the target material, produce a mixture of silver isotopes along with ^111^Ag, namely ^105^Ag, stable silver-107 (^107^Ag), stable ^109^Ag, ^110m^Ag, silver-112 (^112^Ag, *t*_1/2_ = 3.1 h), silver-113 (^113^Ag, *t*_1/2_ = 5.4 h) and silver-115 (^115^Ag, *t*_1/2_ = 20.0 min) [14]. Moreover, ^107^Ag and ^109^Ag are also generated indirectly from the decay of ^107^Cd and ^109^Cd, in turn, deriving from the ^108^Cd(γ,n)^107^Cd and ^110^Cd(γ,n)^109^Cd reactions [14]. As a result, this means of production was not investigated further.

Natural indium has also been considered as a target matrix for the photonuclear production of ^111^Ag through the ^115^In(γ,α)^111^Ag reaction [14]. However, since natural indium is composed of both indium-113 (^113^In, natural abundance 4.29%) and indium-115 (^115^In, natural abundance 95.71%), ^109^Ag is produced alongside ^111^Ag in a 1:18 ratio via the ^113^In(γ, α)^109^Ag reaction [14]. Additional impurities could be produced as well by parallel (γ,n) and (γ,p) reactions on ^115^In, namely indium-114m (^114m^In, *t*_1/2_ = 49.5 d) and stable cadmium-114 (^114^Cd) [14]. Moreover, indium-112m (^112m^In, *t*_1/2_ = 20.7 min) and stable cadmium-112 (^112^Cd) might also be obtained via ^113^In(γ,n) and ^113^In(γ,p) reactions. The presence of these latter radioisotopes of indium and cadmium do not pose an unsolvable issue as they can be removed by post-production chemical purification; however, this way of production is far from being selective for ^111^Ag and so it is difficult to be pursued.

A summary of the energy thresholds for the photonuclear-based reactions induced on natural cadmium and indium targets is gathered in Table 3.

## 3. Production of Silver-103 and Silver-104m,g

As mentioned before, ^111^Ag already represents a theranostic radionuclide, but might also be paired with the positron emitter ^103^Ag and ^104m,g^Ag analogues, in order to provide patients’ selection before treatment by obtaining high-resolution PET images. Unfortunately, the practical applications of these latter radionuclides are still scarcely explored, and their production has mainly been studied only from a theoretical point of view.

The following paragraphs revise the reports concerning the pathways to produce ^103^Ag and ^104m,g^Ag, underlining the potential complexity and practical drawbacks.

### 3.1. Silver-103

**Figure 5:**
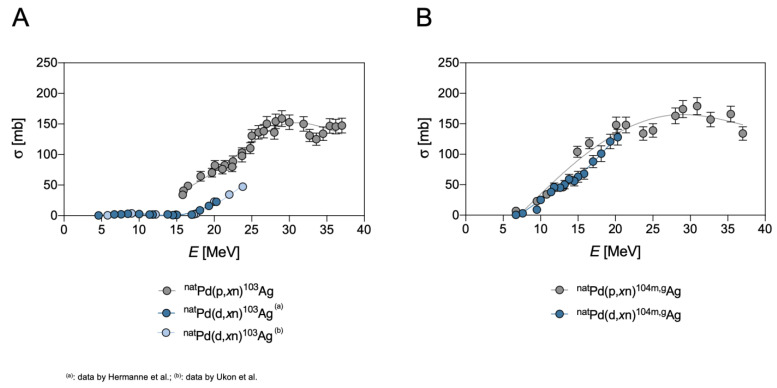
(**A**) Experimental cross-sections of proton-induced ^nat^Pd(p,*x*n)^103^Ag and deuteron-induced ^nat^Pd(d,*x*n)^103^Ag reaction, as derived from Hermanne et al. and Ukon et al. [4,19]. (**B**) Experimental cross-sections of ^nat^Pd(d,*x*n)^104m,g^Ag and ^nat^Pd(p,*x*n)^104m,g^Ag reactions, as derived from Hermanne et al. [3].

^103^Ag can be theoretically produced by a variety of particle-induced reactions, such as the proton irradiation of natural palladium targets via several (p,*x*n) reactions, namely ^104^Pd(p,2n)^103^Ag, ^105^Pd(p,3n)^103^Ag and ^106^Pd(p,4n)^103^Ag, or by the deuterons irradiation of a natural palladium target through the reactions ^102^Pd(d,n)^103^Ag and ^104^Pd(d,3n)^103^Ag, respectively [19]. The cross-sections of these pathways are reported in Figure 5. However, production by proton irradiation is hindered by the presence of the other palladium isotopes composing the target that also induce lower energy (p,n) and (p,2n) reactions such as, for instance, ^105^Pd(p,n)^105^Ag and ^106^Pd(p,n)^106m^Ag.

As already mentioned for the production of ^111^Ag, the radionuclidic impurities of the same element cannot be separated from the sought radionuclide by chemical methods, so the use of a ^104^Pd-enriched target appears to be the only possibility to produce ^103^Ag without longer-life Ag contaminants. Unfortunately, due to the comparable cross-sections, the co-production of ^104m,g^Ag cannot be avoided even in this case and this outcome poses a serious drawback to the possibility of obtaining ^103^Ag with high purity using irradiation with protons. Analogously, it can be observed (Figure 5A) that deuteron-induced reactions to obtain ^103^Ag exhibit such low cross-sections [4], in comparison to the reactions producing ^104m,g^Ag, that the effective exploitation of this pathway is also limited. The cross-sections of the pathways for the production of ^104m,g^Ag are reported in Figure 5B.

**Figure 6:**
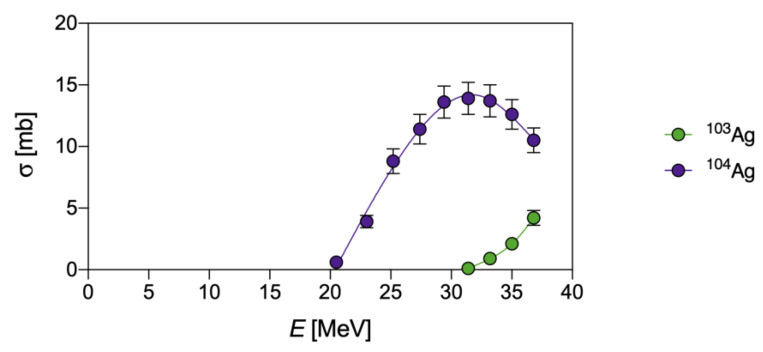
Experimental cross-section of α-induced ^nat^Pd(α,p*x*n)^103^Ag and ^104m,g^Ag, reactions as derived from Hermanne et al. [18].

The production of ^103^Ag is theoretically feasible by α-induced reactions as well, specifically through the reactions prompted on ^102^Pd (Figure 6). However, due to the low natural abundance of this isotope in natural palladium, only highly enriched ^102^Pd targets would ensure a meaningful production of ^103^Ag [18]. Moreover, it is worth mentioning that the contamination with ^104^Ag remains a concern for this means of production, since the reactions yielding the latter radionuclide exhibit lower thresholds in the activation energy and higher cross-sections than the reactions allocated to the production of ^103^Ag on the same target (Figure 6) [18].

Finally, the cross-sections of the proton-induced reaction on the enriched ^104^Pd target, and the α-induced reaction on the enriched ^102^Pd target, reach almost comparable values (up to 800 mb and 500 mb, respectively) [18] and both methods exhibit a limited useful energy span due to the co-production of the aforementioned impurities. However, as the penetration depth is much higher for protons than for α-particles, a larger amount of target material can be irradiated in the first case, achieving significantly higher overall yield. As a result, proton-induced reactions generally prove to be superior to α-induced reactions in the production of ^103^Ag.

### 3.2. Silver-104m,g

As already mentioned in the previous paragraph, ^104m,g^Ag can be produced by irradiating highly enriched ^104^Pd-targets with protons [3]. Unfortunately, the energy span and the cross-sections to produce these radionuclides by the (p,*x*n) pathway almost overlap with those for producing ^103^Ag. Thus, the contemporary production of ^103^Ag, along with ^104m,g^Ag, cannot be avoided, with the only exception of the energy ranging from 5 to 15 MeV, where the formation of ^103^Ag is negligible but, also, the yield in ^104m,g^Ag is relatively low.

^104m,g^Ag can also be obtained from the same target by using deuterons-induced reactions but, in this case, the contamination due to the presence of ^105^Ag, generated by the ^104^Pd(d,n)^105^Ag reaction, cannot be prevented [3]. Even if the values of their cross-sections are similar, the ^104^Pd(p,n)^104m,g^Ag reaction on targets of 500 mg/cm^2^, and with incident energy < 15 MeV, must be preferred over the ^104^Pd(d,2n)^104m,g^Ag reaction [3,20], as the yields for the proton-induced reaction are higher than the deuterons-induced one due to the protons’ longer range through the target. The cross-sections of protons- and deuterons-induced reactions for the production of ^104m,g^Ag are reported in Figure 5B.

The production of ^104m,g^Ag through α-induced reactions on a palladium target is also feasible and the cross-sections for these reactions are reported in Figure 6. The main contribution to this pathway is due to the (α,2n) reaction on the low abundance ^102^Pd. However, considering the fact that the cross-sections are comparable to the (p,n) reactions at a maximal incident energy of 15 MeV, that the energy span is vaguely favorable for protons and that the number of target atoms is higher due to the deeper penetration, the proton-induced reactions on a ^104^Pd target must be preferred over the α-induced ones [18].

**Figure 7:**
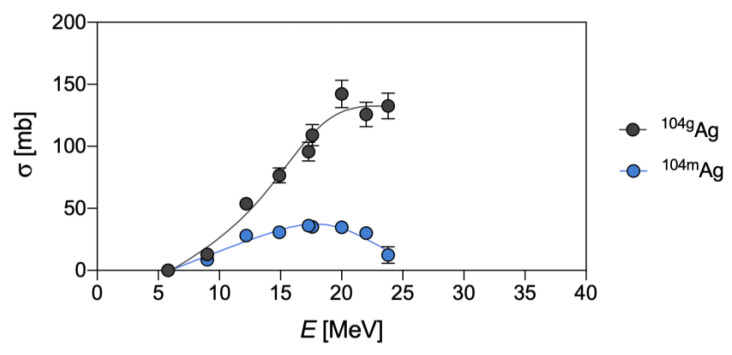
Experimental independent cross-sections for the ^nat^Pd(d,x)^104g^Ag and ^nat^Pd(d,x)^104m^Ag reactions, as derived from Ukon et al. [4].

Finally, the co-production of the metastable state (^104m^Ag), along with the ground state (^104g^Ag), for all the pathways herein summarized, could be a possible drawback for quantitative PET imaging since ^104m^Ag provides a relatively high β^+^ contribution and has a shorter half-life than ^104g^Ag. The independent cross-section determination and evaluation of single contributions are cumbersome tasks and were elucidated, only in the production via the (d,x) reaction, by Ukon et al. [4] (Figure 7).

## 4. Separation of Silver Radioisotopes

### 4.1. Separation by Chemical Methods

The separation step is of utmost importance in the production route of any radiometal for radiopharmaceutical applications, as metallic impurities might compete in the radiolabeling reaction (i.e., with the molecule responsible for the metal binding). Moreover, the presence of unsought radioactive species in the radiometal solution must be strictly avoided since they could deliver an additional unintended dose upon injection. A wide plethora of separation strategies has been developed in recent decades in order to deplete the level of metal impurities and the presence of the target matrix from the produced radioactive silver. In the following paragraph, these separation methods are reviewed and critically analyzed.

#### 4.1.1. Separation of Silver-111 from Palladium Targets

##### Chromatographic Methods

- Cation Exchange: Mansur et al. reported a cation-exchange-based chromatographic separation of ^111^Ag from the neutron-irradiated Pd matrix [21]. According to their protocol, the Pd target (100 mg) was dissolved in aqua regia (5 mL), followed by evaporation to dryness [21]. The process was repeated by adding HCl to remove residual amounts of HNO_3_ and the bulk was dissolved in distilled water (10 mL). Concentrated NH_3_ (25%, 7–8 mL) was then slowly added, and the resulting solution was warmed and passed through a column (1 cm diameter × 10 cm) filled with AG50W-X8 (50–100 mesh, H^+^ form, 5 g) pre-washed with water. A 1 mL/min flow rate was used throughout the process [21]. Silver and palladium cations were therefore adsorbed as [Ag(NH_3_)_2_]^+^ and [Pd(NH_3_)_4_]^2+^ complexes. After a washing step with water (20 mL), necessary to remove the excess of NH_3_, ^111^Ag-containing residue was eluted with a 0.5 M NaCl solution (16 mL, 80% yield) as [AgCl_3_]^2−^. Palladium was recovered by eluting the resin with 14 M HNO_3_ (80 mL). The concentration of Pd^2+^ in the ^111^Ag^+^ eluate was <1 μg/mL.

**Figure 8:**
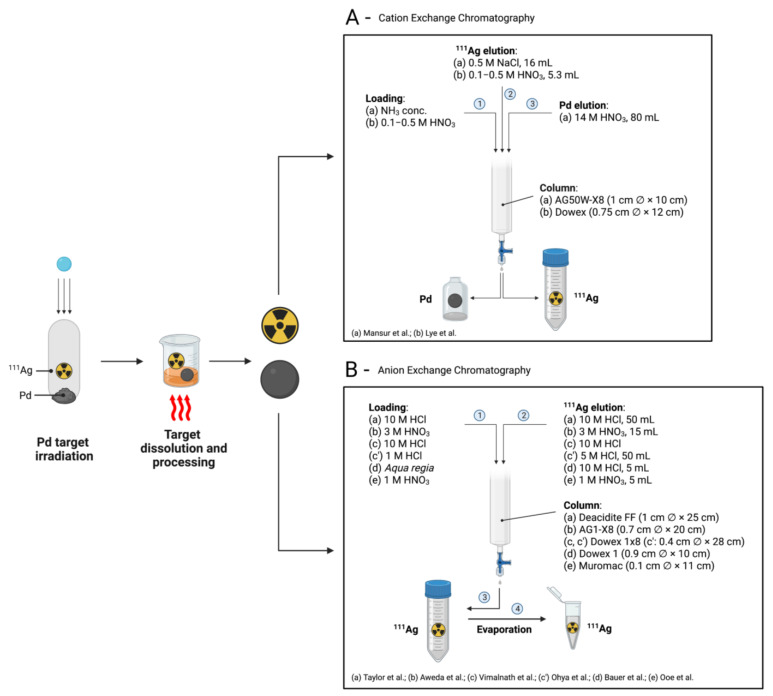
Schematic illustration of (**A**) cation-exchange-based and (**B**) anion-exchange-based chromatographic separation methods of ^111^Ag from irradiated Pd targets [9,10,21,22,23,24,25,26,27]. Image created with BioRender.com.

In another method, reported by Lyle et al., a Dowex chelating ion exchange resin was adopted [22]. In their protocol, the irradiated Pd-target was dissolved in hot HNO_3_ and evaporated to near dryness. The concentrate solution was diluted with water to obtain a 0.1–0.5 M HNO_3_ concentration range and passed through a Dowex resin Al (50–100 mesh, H^+^ form) packed in a column (0.75 cm diameter × 12 cm) [22]. ^111^Ag^+^ was not retained by the resin and was eluted with 0.1–0.5 M HNO_3_. Over 95% of the ^111^Ag^+^-amount could be eluted in one column volume (5.3 mL) and complete elution was obtained after 2–3 column volumes. No measurable ^109^Pd or ^103^Pd contamination was reported by the authors. A schematic depiction of the cation-exchange-based separation processes, described by Mansur et al. and Lyle et al., is shown in Figure 8A.

- Anion Exchange: Taylor et al. reported an anion-exchange-based separation [23]. After the irradiation (4–6 days, neutron flux = 10^12^ n/cm^2^/s), the palladium target was dissolved in aqua regia, and the resulting solution was evaporated to dryness. After heating, the residue was dissolved in a small volume of 10 M HCl to remove traces of HNO_3_ and passed into a column (1 cm diameter × 25 cm) filled with a Deacidite FF anion exchange resin. ^111^Ag-chloro complexes were eluted from the column with a subsequent rinse with 10 M HCl (50 mL). The solution was then evaporated to dryness and the residue dissolved in diluted HNO_3_, recovering 75% of the starting activity.

An alternative method, based on anion exchange resin, was reported by Aweda et al. [9,10]. In this work, a neutron-irradiated Pd-target (4–6 mg) was dissolved in a 1:1 mixture of concentrated HCl and HNO_3_ (4 mL). The resultant solution was heated to near dryness and reconstituted with 3 M HNO_3_ (0.5–0.65 mL) twice to expel traces of HCl [9,10]. Additional 3 M HNO_3_ (2 mL) was then added. The mixture was loaded into an AG1-X8 anion exchange resin (4.7 g, 0.7 cm diameter × 20 cm) preconditioned with 3 M HNO_3_. In these conditions (and generally in HNO_3_ concentrations higher than 1 M), Pd^2+^ forms anionic complexes such as [Pd(H_2_O)(NO_3_)_3_]^−^ and [Pd(NO_3_)_4_]^2−^, which are strongly retained by the AG1-X8 resin, while Ag^+^ remains in a cationic form that is weakly absorbed. ^111^Ag^+^ was hence eluted in 3 M HNO_3_ (15 mL) and several fractions were combined and dried down under air and gentle heating. The average ^111^Ag^+^ recovery was 92.9 ± 23.7% with a Pd^2+^ concentration <25 ppb.

Vimalnath et al. described the use of a Dowex 1X8 anion exchanger column [24]. In their method, the processed target was obtained in a 10 M HCl solution and the mixture containing palladium and ^111^Ag was loaded into the column. Under these conditions, Pd^2+^ was strongly retained by the resin while ^111^Ag^+^ was easily eluted out. Recently, Ohya et al. studied the separation of the silver radioisotopes obtained from proton- or deuteron-irradiated ^nat^Pd-targets by using the same anion exchange resin (Dowex 1X8) [25]. The irradiated target was dissolved in aqua regia (10 mL) at 110 °C, and then evaporated to dryness. A few mL of concentrated HCl was then added and evaporated to dryness to eliminate the HNO_3_ residue (this process was repeated four times). After evaporation, the precipitate was redissolved in 1 M HCl (8 mL) and the solution was loaded onto the anion-exchange resin (0.4 cm diameter × 28 cm). Subsequently, 1 M HCl (90 mL) was flushed to remove the rhodium radioisotopes while 5 M HCl (50 mL) was loaded to elute the silver ones. The authors reported that the elution with 5 M HCl was preferred to the previously reported use of 10 M HCl by Vimalnath et al. [24] as traces of palladium were found in the fractions containing radioactive silver when the more concentrated acid was employed. The final yield was 98% with silver almost quantitatively contained in a 20 mL fraction. Finally, concentrated HCl (45 mL) was loaded into the column to recover palladium. All elution procedures were performed using a flow rate of 0.5 mL/min.

A further alternative anion-exchange-based method was reported by Bauer et al. [26]. In their work, Pd was irradiated with neutrons for 7 d at the 10 MW reactor at the research center Risø in order to obtain 400 MBq of ^111^Ag and a roughly equal activity of ^109^Pd. The palladium foil was dissolved in aqua regia (1 mL) and the solution was passed through a Dowex 1 strong anion exchange column (0.9 cm diameter × 10 cm) previously equilibrated in 10 M HCl. ^111^Ag was eluted in 10 M HCl (5 mL) and then evaporated to 50 μL in a glass tube. It is worth underlining that the author reported a 50% activity loss, as half of ^111^Ag resulting activity was fixed in the glass beaker during the transfer. No trace of Pd (monitored as ^109^Pd activity) was detected in the ^111^Ag fraction. The same protocol was recently employed by Tosato et al. in order to produce ^111^Ag, which was subsequently used for radiolabeling experiments [6,7].

Finally, Ooe et al. recently reported the production of ^111^Ag via 24-MeV deuteron beam irradiation of ^nat^Pd foils [27]. After irradiation, the Pd target was dissolved in a mixed HNO_3_ and HCl solution and then evaporated to dryness. The residue was dissolved in 1 M HNO_3_ solution and passed through a Muromac anion exchange column (200–400 mesh, NO_3_^−^ form, 0.1 cm diameter × 11 cm). ^111^Ag was eluted in 1 M HNO_3_ (5 mL, recovery yield 99%) while Pd was stripped using concentrated HNO_3_ (recovery yield 98%). A schematic illustration of the anion-exchange-based separation processes described herein is shown in Figure 8B.

- Alumina Adsorption: Khalid et al. reported the possible use of alumina to adsorb the produced ^111^Ag and separate it from a neutron-irradiated palladium bulk matrix [28]. In their work, the irradiated Pd target (100 mg) was dissolved in aqua regia (5 mL) and the solution was evaporated to dryness. Repeated additions of concentrated HCl were performed to expel traces of HNO_3_ and the evaporation was carried out again. Then, the obtained residue was dissolved in 0.01 M HCl (30 mL) and the solution was passed at 1 mL/min through an alumina-containing column (5 g, 1 cm diameter × 10 cm) pre-conditioned with 0.01 M HCl. The column was washed with 0.1 M HCl (60 mL) to remove Pd^2+^ and the ^111^Ag-labelled residue was eluted with 4 M HCl (30–40 mL). More than 80% of the ^111^Ag-fraction was recovered in 20 mL and the palladium concentration was estimated to be <1 μg/mL.

##### Liquid/Liquid Extraction

Alberto et al. reported the possibility of using liquid/liquid extraction to separate ^111^Ag-containing fractions from neutron-irradiated Pd-matrices [13]. According to this procedure, an irradiated natural palladium target (100 mg) was refluxed in concentrated HNO_3_ (10 mL) until complete dissolution. Subsequently, the sample volume was halved by distillation and the solution was diluted with water (35 mL). The liquid/liquid extraction was performed by mixing a part of the sample (25 mL) with toluene (75 mL) and stirring it at 400 rpm. The addition of 0.089 M triphenylphosphine (TPP) in toluene (1.2 mL) allowed the recovery of the radioactive fraction containing ^111^Ag into the organic phase [13]. On the other hand, palladium remained almost entirely in the aqueous phase as TPP-complex or precipitated as [Pd(TPP)_2_(NO_3_)_2_]. After 25 min, the organic phase was removed and filtered (via an inorganic Anotop 0.2 μm filter). The addition of 0.1 M acetate buffer (5 mL, pH 6) allowed the re-extraction of ^111^Ag^+^ at a >70% yield. The residual amount of Pd^2+^ was determined by inductively coupled plasma mass spectrometry (ICP-MS) and resulted in a depletion by a factor of up to 27,000 (~3 μg).

Lahiri et al. reported the production of ^103^Ag, ^104^Ag, ^105^Ag, ^106^Ag, ^110^Ag and ^112^Ag (along with ^101^Rh, ^105^Rh and ^106^Rh and ^104^Cd, ^105^Cd, ^107^Cd, ^109^Cd and ^111^Cd) by the α-particle irradiation of a natural palladium target in a 40 MeV cyclotron (2 μA), and their subsequent extraction and separation by using di-(2-ethylhexyl)phosphoric acid (HDEHP) as a liquid cation exchanger [29]. The activated palladium target was dissolved in 4 M HNO_3_ and the solution was made ammoniacal by the addition of concentrated NH_3_ followed by the addition of H_2_O_2_. The solution was then shaken with an equal volume of 0.1% HDEHP/octanol solution. Cadmium radioisotopes were extracted by the organic phase due to the formation of cationic cadmium species like [Cd(OH)(H_2_O)_x_]^+^ and [Cd_2_(OH)(H_2_O)_x_]^3+^, leaving rhodium and silver radioisotopes, along with the palladium of the matrix, in the aqueous phase. The aqueous phase was treated with an equal volume of 10% HDEHP/0.1 M citric acid solution and the silver radioisotopes, along with the bulk palladium, were completely extracted as [Pd(H_2_O)_4_]^+^ and [Ag(H_2_O)_2_]^+^, respectively. Finally, the silver radioisotopes were stripped into 6 M HNO_3_ [29].

Lahiri et al. also developed another process using trioctylamine (TOA) as a liquid anion exchanger to separate ^103^Ag, ^104^Ag, ^105^Ag, ^106^Ag, ^110^Ag and ^112^Ag from ^101^Rh, ^105^Rh and^106^Rh and ^104^Cd, ^105^Cd, ^107^Cd and ^109^Cd from an α-particle-activated palladium target (40 MeV, 2.3 μA) [30]. After the target dissolution in 0.1 M HNO_3_, the aqueous phase was mixed with an equal volume of organic extractant (0.1 M TOA). Palladium was quantitatively extracted in the organic phase as [Pd(NO_3_)_6_]^2−^ anionic complex, leaving the Cd, Rh and Ag radioisotopes in the aqueous phase. After palladium separation, the aqueous solution was converted to a 5 M HCl solution and Cd (100%) and Ag (90%) radioisotopes were extracted as anionic chloro-complexes by the addition of TOA. Contamination with Rh was < 5%. The extraction with 8 M HCl stripped back the Ag radioisotopes into the aqueous phase and they could be separated by Cd ones.

**Figure 9:**
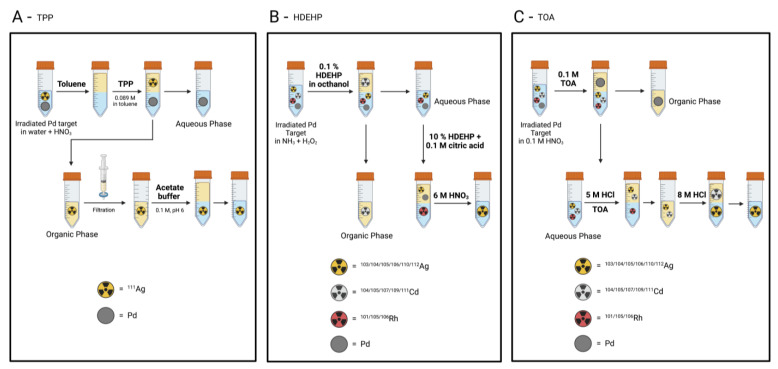
Graphical illustration of liquid/liquid extraction processes for the separation of ^111^Ag from Pd targets [13,29,30]. Image created with BioRender.com.

A schematic illustration of the liquid/liquid extraction processes herein described is shown in Figure 9.

##### Precipitation

- Precipitation as silver chloride: Collin et al. reported the dissolution of an irradiated palladium wire in hot concentrated HNO_3_ spiked with one drop of HCl [31]. Radioactive silver was then co-precipitated with an amount of stable AgNO_3_, inserted to increase the total mass of silver, by the addition of NaCl. Filtration allowed the recovery of AgCl, which was then dissolved in NH_3_ solution and reduced back to metallic silver by using ascorbic acid.

An alternative precipitation-based process was reported by Sicilio et al. [32]. In their study, 1 g of reactor-irradiated palladium was dissolved in aqua regia (8 mL) and the solution was heated at 65 °C for 45 min. Concentrated HCl was then added (5 mL), along with an inactive silver carrier, prepared by adding 10 mL of concentrated HCl to 19 mg of AgNO_3_ dissolved in 1 mL of water [32]. Silver chloride was precipitated by dilution with water (2 L) overnight at room temperature and then filtered. AgCl was washed with 1% HNO_3_ to avoid peptization, re-dissolved in concentrate NH_3_ and then precipitated again as chloride by the addition of 1–2 drops of HCl. The solution was made acidic with 6 M HNO_3_ and further reprocessed by using the protocol described above. The final product was obtained with an overall yield of 90% in concentrate NH_3_ (3 mL) and the palladium content was reported as being less than a few μg [32].

Further processes involving the precipitation of ^111^Ag were reported in the pioneering work of Zimen et al. who, after the dissolution of the palladium target in aqua regia and dilution with 100 mL water, precipitated ^111^Ag along with a silver nitrate carrier as ^111^AgCl and separated it from the palladium in the solution [33]. After a process of purification and dissolution involving the addition of hot NH_3_ followed by re-precipitation with HCl, ^111^AgCl was reduced to metallic silver using a H_2_ flow at 500 °C and finally reconstituted in HNO_3_.

Very recently, Blackadar et al. employed precipitation processes to isolate ^111^Ag from neutron-irradiated palladium targets [8]. The palladium foil (1.18 mg) and ^111^Ag were first dissolved and oxidized to Pd^2+^ and ^111^Ag^+^ in a 1:1 mixture of concentrated HNO_3_ and HCl (500 μL). Concentrated HNO_3_ (2 mL) was added, and the solution was gently evaporated to expel HCl (final volume 800 μL). The obtained solution was diluted with water (300 μL) and a 0.118 M carrier solution of AgNO_3_ (50 μL), along with phosphate-buffered saline (PBS, 200 μL), was added to induce the precipitation of ^111^Ag^+^ as AgCl. The solid AgCl was then easily isolated from the highly soluble PdCl_2_ and was re-dissolved in ammonium hydroxide (200 μL) to generate [Ag(NH_3_)_2_Cl] [8]. The procedure was performed in less than 1 h with a ^111^Ag recovery of 93% and a palladium removal of >99.99%.

**Figure 10:**
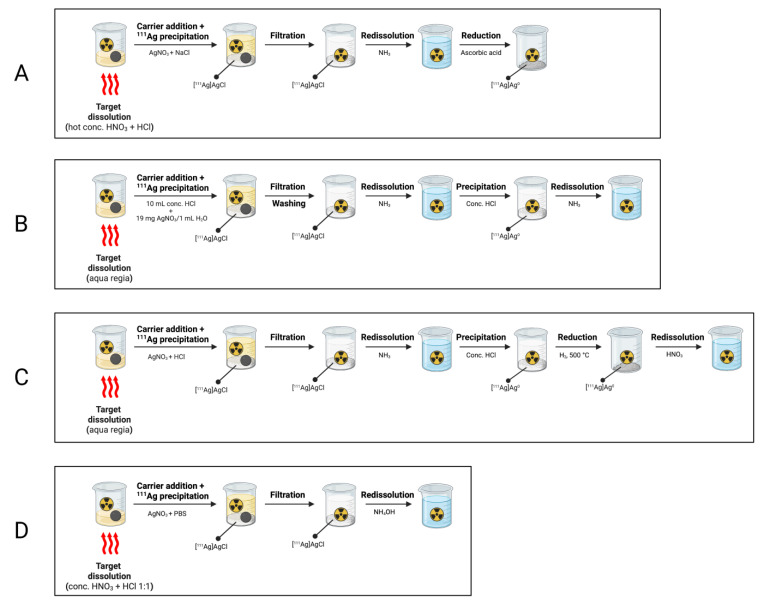
Schematic representations of precipitation-based processes for the separation of ^111^Ag from Pd targets proposed by (**A**) Collin et al., (**B**) Sicilio et al., (**C**) Zimen et al. and (**D**) Blackadar et al. [8,31,32,33]. Image created with BioRender.com.

Figure 10 summarizes all the precipitation-based processes herein reported.

- Co-precipitation with mercury(I) chloride: Haymond et al. reported the separation of radioactive silver radioisotopes (^105^Ag, ^106^Ag and ^111^Ag) produced by bombarding a palladium target with 19 MeV deuterons (200 μA/h, average beam intensity 20 μA) through a precipitation technique using mercury(I) chloride as co-precipitant [16]. After the bombardment, the irradiated surface of the palladium target (approximately 0.5 g of Pd) was milled off and dissolved in aqua regia. The solution was evaporated to dryness and dissolved in 0.5 M HCl (500 mL) containing 50 mg of rhodium and ruthenium hold-back carrier. Then, a saturated solution of HgNO_3_ (0.5 mL) was added and the mixture was vigorously stirred. The precipitate of Hg_2_Cl_2_, containing over 95% of the radioactive silver, was centrifuged, washed with 0.5 M HCl and dissolved in the minimum needed volume of 16 M HNO_3_ [16]. Na_2_SO_4_ (200 mg) was then added, and the solution was evaporated to dryness (450 °C, 2 h) to drive off the mercury carrier. The residue was quantitatively solubilized in distilled water (10 mL) to give an isotonic saline solution of radioactive silver.

##### Co-Crystallization

Micheev et al. demonstrated that ^111^Ag can be separated from neutron-irradiated palladium targets by combined co-crystallization with NaCl and electrodeposition [34]. In fact, as NaCl and AgCl are isomorphous compounds, ^111^Ag^+^ can replace a Na^+^ ion in the crystal lattice of NaCl crystals, thus being quantitatively separated from the Pd^2+^ that remains in the solution. In the described method, the irradiated Pd target (1 mg) was treated with aqua regia (5 mL) and the obtained solution was evaporated to dryness. HCl was added and subsequently removed by evaporation [34]. The residue was dissolved in a saturated NaCl solution (50 mL) at 50 °C and the mixture was filtered with a G3 sintered glass disk. After 3 h of continuous stirring about 45% of NaCl, containing more than 95% of ^111^Ag^+^ trapped in the lattice, was separated as solid crystals. Crystals were filtered and washed with saturated NaCl (3 × 10 mL) to remove Pd^2+^ traces. In the right conditions, it was shown that traces of co-precipitated Pd^2+^ do not exceed 0.01% of the separated ^111^Ag^+^ activity. Finally, the authors stated that the saline solution containing ^111^Ag^+^ can be directly used for medical preparations or, alternatively, it can be readily separated by electrodeposition on a Pt electrode and subsequently dissolved in HNO_3_ [34].

##### Electrodeposition

Griess and Rogers obtained carrier-free ^111^Ag by electrolysis of the palladium target solutions [35]. Their method entailed the incorporation into aqueous palladium solutions of a stoichiometric excess of ancillary compounds able to form water-soluble complexes with both Ag^+^ and Pd^2+^ (e.g., NH_3_, cyanides, thiocyanates, thiosulfates). The solution was kept at an alkaline pH and then subjected to electrolysis to attain the ^111^Ag^+^ electrodeposition upon a Pt cathode [35]. Practically, the neutron-irradiated natural palladium target was dissolved in a 1:10 mixture of hot concentrated HNO_3_ and HCl (50 mL/g Pd). The nitrate ion was then removed by fuming and the excess acid was completely neutralized by adding a solution of NaOH. The complexing agent was added to this solution and the mixture underwent several cycles of electrolysis at ambient temperature. Then, a large part of the solution was removed by suction and the cell was carefully flushed with 0.1 M sodium perchlorate and water. Finally, the cathode was removed and dried for the subsequent recovery of the electroplated ^111^Ag. Under the proper electrolysis conditions and using 0.1 M NaCN + 1.0 M NaOH as complexing agents, a ^111^Ag recovery around 77% was reported.

##### Other Techniques

Selective adsorption on platinum surface: Miller et al. reported the possibility of separating traces of radioactive silver from palladium solutions through the selective adsorption of the silver ions on a platinum surface [36]. In the given case, the concept of “adsorption” covers an exchange reaction between the hydrogen atoms bound to the platinum surface and the silver ions in the solution. The method is based on the phenomenon that ions present in micro-concentration may show different behavior in many aspects from the same ions in macro-concentration [36]. This is the case for silver and palladium. Practically, irradiated palladium (0.1 g) was dissolved in aqua regia, and the obtained solution was evaporated to dryness. After driving off any trace of HCl, the residue was dissolved in 0.001 M HNO_3_. Preliminary anodic or cathodic polarized platinum wire was used as an adsorbent and was immersed into the solution and kept under constant stirring. After 30 min, 95–97% of ^111^Ag^+^ were found to be adsorbed by the platinum wire. The desorption proved to be completed after having rinsed the adsorbent with a small volume of 9 M HNO_3_ [36].

#### 4.1.2. Separation of Silver-111 from Other Targets or Contaminants

**Figure 11:**
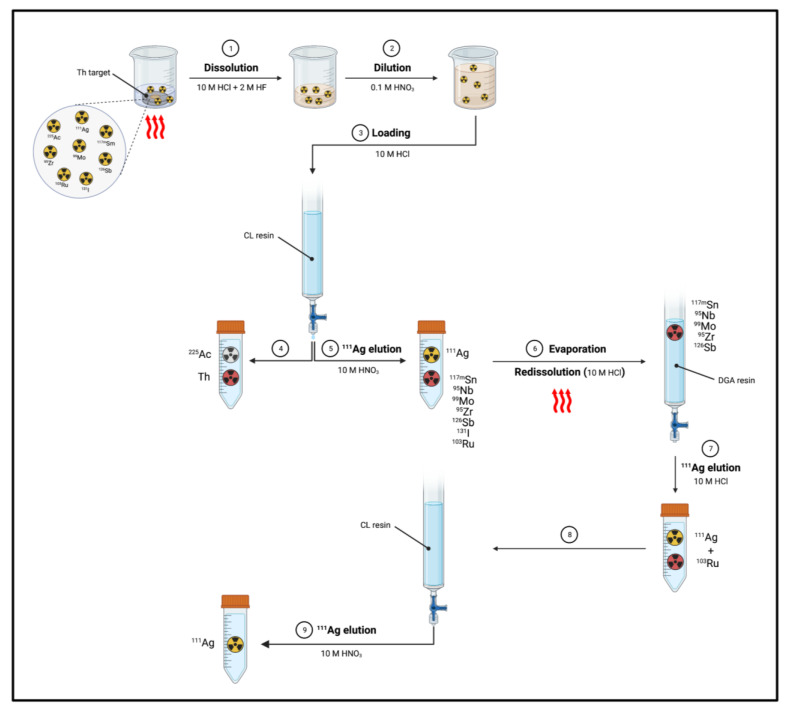
Separation of ^111^Ag from proton-irradiated Th target according to method 1 [1]. Image created with BioRender.com.

- Separation of ^111^Ag from Th target: Mastren et al. reported the recovery of ^111^Ag from a proton-irradiated thorium matrix using a solvent-impregnated CL resin composed of alkyl phosphine sulfides and alkyl phosphine oxides [1]. The author proposed two methods, both of them involving the dissolution of the irradiated thorium target (10 g) in a mixture of 10 M HCl (200 mL) and 2 M HF (0.1 mL) by heating at 80–90 °C for 2 h. Then, in the first method (Figure 11), an aliquot of the dissolved target (0.1 mL) was diluted with 0.1 M HNO_3_ (5 mL). Subsequently, 50 μL of the obtained solution was diluted with 10 M HCl (5 mL) and passed through a column containing the preconditioned (10 M HCl) CL resin (1 mL). ^225^Ac produced from irradiation and the bulk Th target were not retained by the resin, whilst ^111^Ag was blocked and then eluted by using 10 M HNO_3_ (5 mL).

The process gave a 94% yield but a 79% radionuclide purity, since many radioactive contaminants co-eluted with ^111^Ag^+^, i.e., ^117m^Sn (6%), ^95^Nb (6%), ^99^Mo (3%), ^95^Zr (2%), ^126^Sb (2%), ^131^I (1%) and ^103^Ru (1%). Thus, the ^111^Ag-containing fraction underwent a subsequent fine-purification step. After evaporation and reconstitution with 10 M HCl (5 mL), the solution was passed through a DGA (*N*,*N*,*N*′,*N*′-tetra-*n*-octyldiglycolamide) extraction chromatography column (1 mL). Under these conditions, ^117m^Sn, ^95^Nb, ^99^Mo, ^95^Zr and ^126^Sb were highly adsorbed and could be separated, while the solution (10 M HCl, 3 mL) was further passed through a CL resin (250 μL) and subsequently washed with 10 M HCl (2 mL) for extracting ^103^Ru. Finally, ^111^Ag was obtained by flushing 10 M HNO_3_ (5 mL) through the resin. ^111^Ag recovery by this process was reported as being equal to 93% with a radiochemical purity of >99.9%.

**Figure 12:**
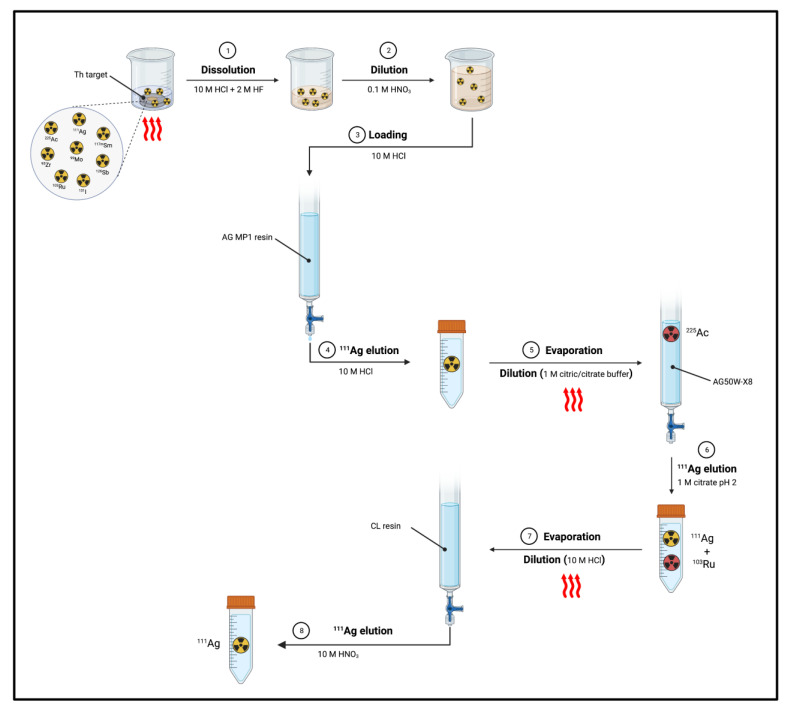
Separation of ^111^Ag from proton-irradiate Th target according to method 2 [1]. Image created with BioRender.com.

In the second process (Figure 12), the dissolved target was passed through an AG MP1 resin (15 mL, 100–200 mesh, chloride form) [1]. The eluate and the subsequent washing solution (10 M HCl, 15 mL) containing ^111^Ag was evaporated to near dryness and diluted with 1 M citric acid/citrate buffer (750 mL, pH 2). The solution was then passed through a cation exchange column containing AG50W-X8 (15 mL) and two washing steps with 1 M citrate pH 2 (5-column volume) were followed. ^225^Ac was retained by the column while ^111^Ag was recovered in the washing solution.

The process removed the greatest part of the radionuclidic impurities from the ^111^Ag solution but a ^103^Ru contamination (<1%) was still present. The latter was hence removed by passing the solution, previously evaporated to dryness and reconstituted with 10 M HCl (5 M), through a CL resin column (250 μL). The column was then washed with 10 M HCl (2 mL) and ^111^Ag was eluted with 10 M HNO_3_ (5 mL) [1]. The total recovery yield was 89% with a radiochemical purity of 99%.

Despite the potential of producing ^111^Ag concomitantly with ^225^Ac, the time-consuming separation of the two radionuclides, as well as the presence of not negligible amount of ^110m^Ag (*t*_1/2_ = 249.8 d), represent the disadvantages of this method [1].

- Separation of ^111^Ag from ^111^Cd: Stable ^111^Cd is the main isobaric contaminant remaining when ^111^Ag is obtained via the isotope mass separation on-line technique (ISOL—vide infra). To efficiently separate Ag^+^ from Cd^2+^ and selectively harvest ^111^Ag, Tosato et al. employed an extraction chromatographic resin (CL resin) [37] and developed three alternative separation methods. In the first one, upon the loading of the resin, Cd^2+^ was quantitatively removed in the washing step (0.1 M HNO_3_) whereas Ag^+^ was firstly retained and subsequently eluted with 10 M HNO_3_. The reported recovery yield was 90 ± 5% [37]. In the second separation method, 7 M NH_3_ was used to quantitatively elute Ag^+^ instead of 10 M HNO_3_. Finally, 1 M H_2_SO4 was employed in the washing step of the third method to selectively remove Cd^2+^, while the elution of Ag^+^ was conducted using 0.1 M thiourea (yield 92%). Although these methods hold promise in depleting Cd contamination from ISOL-produced ^111^Ag, they have yet to be tested using irradiated samples.

### 4.2. Mass Separation Method

The mass separation technique evolved from chemical techniques used to separate radioactive isotopes from irradiated targets. According to this method, the radionuclides of interest are produced by irradiating a target with a particle beam (e.g., protons). Then, the produced radioactive species are ionized and released from the target. Subsequently, the so-generated ion beam is extracted and directed to a magnet where all the concurrently produced non-isobaric species are separated. After the mass-separation step, the isobaric radioactive beam containing the radionuclide of interest is dumped on an implantation substrate for the collection [38,39].

In this context, the Legnaro National Laboratories of the Italian Institute of Nuclear Physics prompted a line of research aimed at the production of ^111^Ag via the ISOL technique in the framework of the ISOLPHARM (“ISOL technique for radioPHARMaceuticals”) project [38,40,41]. Given that the facility is currently under construction, only theoretical calculations and off-line experiments have been conducted so far [38,41]. According to these calculations, ^111^Ag could be produced with elevated production rates and extremely high purity (e.g., in-target production equal to 9.5 GBq after 0.5 d of irradiation and 83 GBq after 5 d of irradiation) by bombarding a uranium carbide target with a 40 MeV proton beam (200 μA) [38,41]. Indeed, after the mass separation stage, only the isobaric contaminant ^111^Cd is expected to be collected along with ^111^Ag [37,38,41], but it can be removed by dedicated chemical processing steps as described in the previous paragraphs [37].

## 5. Final Remarks and Future Perspectives

In the present review, several routes for the production of silver radioisotopes of potential medical interest (i.e., ^103^Ag, ^104m,g^Ag and ^111^Ag) have been revised and discussed. While the production of the dual therapeutic and SPECT-diagnostic ^111^Ag has been extensively explored in the literature, its related PET-imaging counterparts, ^103^Ag and ^104m,g^Ag, have still been scarcely considered. As a result, the production of highly specific activity ^111^Ag could be achieved by the neutron irradiation of a ^110^Pd-enriched target or, alternatively, by low-energy (around 5 MeV) deuteron-induced reactions on the same target. Across both, the first pathway must be preferred due to the higher yield per unit amount of target material. The production of pure ^103^Ag is still an unsolvable concern due to the certain co-production of ^104m,g^Ag, whichever pathway is followed. That being stated, the highest yielding process appeared to be the proton irradiation of ^104^Pd-targets via several (p,*x*n) reactions. Concerning ^104m,g^Ag, it can be theoretically obtained in a low yield but sufficiently high molar activity by irradiating highly enriched ^104^Pd-targets with protons in an energy range from 5 to 15 MeV, where the formation of ^103^Ag is restricted. The independent production of ^104m^Ag and ^104g^Ag is, conversely, thus far inconceivable. An alternative approach to address the lack of sufficiently pure diagnostic isotopes of silver, could be the coupling of the therapeutic ^111^Ag with a diagnostic radionuclide of a different element with sufficiently similar chemical behavior, such as the case of the “improper” matching pair formed by gallium-68 (^68^Ga) and ^177^Lu. Belonging to the same chemical group of silver, the most suitable candidates might be the radioisotopes of copper (such as copper-61, -62 or -64). However, the chemical and coordination properties of Cu^2+^ (which is the species afforded for copper radionuclides) are quite different from those of Ag^+^ (which is the species afforded for silver radionuclides), so such a pairing is barely warranted.

The mass separation technique appears to be able to overcome almost all the problems concerning the co-production of silver radioisotopes related to the routes reported herein [42,43]. Indeed, it allows the separation (either on-line or off-line, i.e., post an external irradiation) and the subsequent collection of the single radionuclide of interest. However, as the production yield is highly dependent on the ionization efficiency, extremely effective ionization processes must be developed to effectively achieve the yields obtained by theoretical calculations. Moreover, facilities owning the technologies to exploit this technique are still very limited around the world.

Regarding the chemical separation methods, sundry studies, mainly restricted to the ^111^Ag production and its relative issues, have been carried out in recent decades. These methods include chromatographic techniques, liquid/liquid extraction, precipitation, co-crystallization and electrodeposition. The chromatographic methods generally allow the proper separation of ^111^Ag from the irradiated palladium bulk and co-produced elements but have the significant drawback of providing ^111^Ag in large volumes of concentrated acidic solutions that are unsuitable for the direct labeling process of biological vectors. Further evaporations and reconstitutions in weakly acidic solutions are hence necessary to comply with the requirement of these molecules. These kinds of processes generally introduce other contaminants into the final solution, thus decreasing the overall yield. As the methods involving precipitation require the co-addition of a macroscopic quantity of stable silver compounds to allow for the efficient separation and recovery of the silver radioisotopes from the irradiated target material, they are not suitable for radiopharmaceutical applications entailing the labeling of targeting vectors. In fact, the addition of carriers would decrease the molar activity of the final product under acceptable thresholds. In the same way, the separation method based on mercury(I) chloride extraction cannot be admissible either, as it likely introduces traces of impurities that would affect the medical safety of the final product. Despite their efficiency, other processes referred to in this section do not seem suitable for radiopharmaceutical production directed to medical applications either, as their automation appears challenging. As a result, an optimal separation process that allows the sourcing of radioactive silver in a formulation that can be directly used for the labeling of biological vectors (i.e., mild-acidic pH, small volume), avoiding the tedious steps of evaporation and re-dissolution, has not been reported to date. The test labelling of chelators with small amounts of ^111^Ag has only been attained up to now [6].

## 6. Conclusions

Although ^103^Ag, ^104m,g^Ag and ^111^Ag are very attractive radioisotopes for personalized medicine, embedding therapeutic and diagnostic potential in a triplet of the same element, research in this field still has many open avenues for the scientific community. Demanding challenges entail reliable and available means of production, as well as proper purification methods able to provide ^103^Ag, ^104m,g^Ag and ^111^Ag in ready-to-use formulations. Among the three radionuclides, ^111^Ag is hitherto the most studied, and several reports regarding its production and purification are already available. The scaling up of the production in order to reach amounts comparable to other radionuclides used for therapy—such as, for instance, ^177^Lu, that exhibits almost equivalent emission properties—appears to be feasible in affordable ways both by traditional methods (i.e., using nuclear reactors) and with high-energy cyclotrons coupled with on-line mass separation. Due to its certain potential, ^111^Ag is one of the novel radionuclides available in the PRISMAP portfolio (https://www.prismap.eu/radionuclides/portfolio/111Ag/ accessed on 10 November 2022) and small amounts of purified radionuclides are possibly provided upon the submission of research projects of mutual interest, aiming to foster the development of this isotope. Moreover, the decay properties of ^111^Ag make it suitable for perturbed angular correlation (PAC) spectroscopy and its possible applications have recently increased the interest of researchers in the field [7]. The production and separation of ^103^Ag and ^104m,g^Ag appears more challenging but, due to the similarity of the half-lives and of the *β*^+^-emissions in terms of energy and abundance, the mutual contamination of the two radionuclides in the same solution should not be a concern and might be potentially used for imaging purposes as well. Due to the shortage of knowledge related to stable Ag-complexes in physiological conditions, deeper chemical and radiolabeling studies, able to provide suitable chelators for preclinical and clinical applications, are mandatory in order to foster the widespread application of these silver radionuclides; however, notable efforts have already been attempted in providing sulfur-rich macrocyclic ligands able to coordinate ^111^Ag with good preliminary outcomes [6].

## Figures and Tables

**Table 2:** Natural abundance of isotopes composing natural Pd and corresponding nuclear reactions activated upon neutron irradiation (5 × 10^13^ n/cm^2^/s neutron flux) [3,14].

Isotope	Natural Abundance [%]	Nuclear Reaction and Final Product	Cross-Section [mb]
Palladium-102	1.02	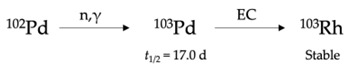	180
Palladium-104	11.14	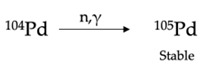	75
Palladium-105	22.33	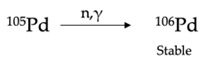	217
Palladium-106	27.33	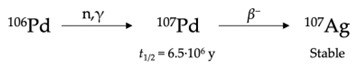	29
Palladium-108	26.46	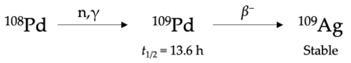	868
Palladium-110	11.72	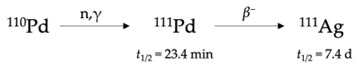	340

EC = electron capture.

**Table 3:** Energy thresholds for the photonuclear-based reactions induced on cadmium and indium targets, as derived from El-Azony et al. [14].

Target Material	Produced Isotope	Threshold Energy [MeV]
Natural Cd	^111^Ag	9.6
	^105^Ag	7.3
	^107^Ag	8.1
	^109^Ag	8.9
	^110m^Ag	9.1
	^112^Ag	9.7
	^113^Ag	10.2
	^115^Ag	11.0
	^107^Cd	10.3
	^109^Cd	9.9
Natural In	^111^Ag	3.7
	^109^Ag	3.1
	^114m^In	9.0
	^114^Cd	6.8
	^112m^In	9.4
	^112^Cd	6.1

## Data Availability

Not applicable.
